# COF RIO-18/Polyurethane Mixed Matrix Membranes with Enhanced CO_2_ Permeability and CO_2_/N_2_ Selectivity

**DOI:** 10.1021/acsomega.6c06026

**Published:** 2026-08-21

**Authors:** Paulo V. S. Campos, Andresa V. Ramos, Alberto C. Habert, Pierre M. Esteves

**Affiliations:** † Instituto de Química, 28125 Universidade Federal do Rio de Janeiro, Av. Athos da Silveira Ramos, 149, CT, A-622, Cid. Univ., Rio de Janeiro, Rio de Janeiro 21941-909, Brazil; ‡ Nanotechnology Engineering Program, COPPE, Universidade Federal do Rio de Janeiro, Rio de Janeiro 21941-972, Brazil; § Chemical Engineering Program, COPPE, Universidade Federal do Rio de Janeiro, Rio de Janeiro 21941-972, Brazil

## Abstract

A beta-ketoenamine-linked covalent organic framework named RIO-18, also reported as TpPa COF, featuring CO_2_-philic C=O/N–H sites and ∼17 Å pores, was incorporated into polyurethane by the casting method at loadings of 0.1–5 wt % to prepare xRIO-18@PU mixed matrix membranes (MMMs). Pure-gas permeation of CO_2_, CH_4_, and N_2_, along with their corresponding ideal selectivities, were evaluated at different feed pressures. Thermal and structural analyses confirm that the COF was successfully incorporated into the matrix without altering the polymer structure. Gas permeation tests revealed a substantial increase in CO_2_ permeability (up to 138%) and CO_2_/N_2_ ideal selectivity (up to 454%) as compared with unloaded PU, with the highest permeability and selectivity gains observed at 3 bar, while CO_2_/CH_4_ selectivity remained comparatively stable across the pressure range studied. These improvements are consistent with preferential CO_2_–COF interactions and additional transport pathways associated with well-dispersed RIO-18 particles, with COF loading strongly affecting permeation. Overall, the results identify CO_2_/N_2_ separation, relevant to postcombustion CO_2_ capture scenarios, as the most promising application for these proof-of-concept MMMs, while mixed-gas permeation, sorption/diffusion analysis, and long-term stability remain to be addressed in future work.

## Graphical abstract

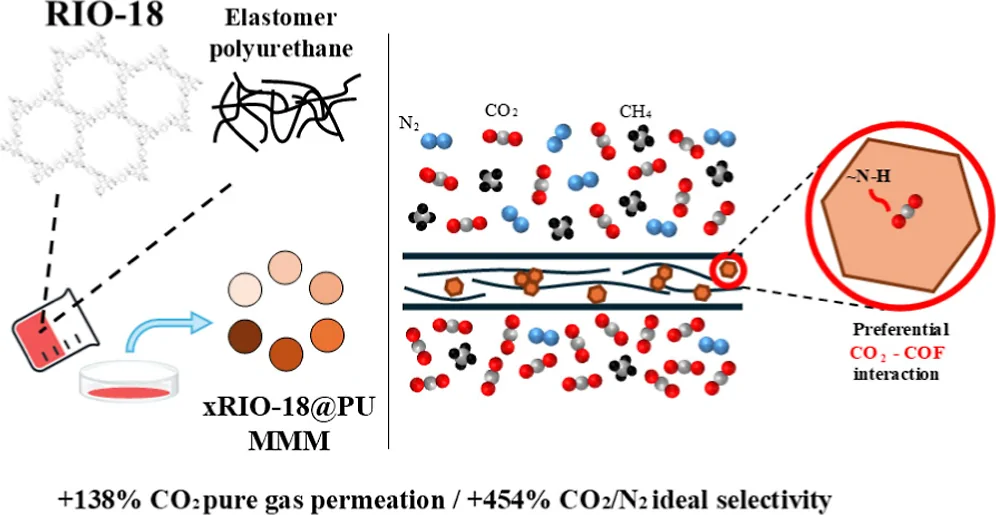

## 1 Introduction

Microporous and mesoporous materials have long played a central role in separation processes due to their high surface areas, well-defined pore structures, and tunable chemical functionalities. Traditionally, inorganic materials such as zeolites and mesoporous silicas have dominated this field. More recently, covalent organic frameworks (COFs) have emerged as a versatile class of crystalline nanoporous materials, composed entirely of light elements linked through strong covalent bonds (e.g., C–C, C–O, and C–N). Their modular design enables precise control over pore size, topology, and functionality, making them attractive for applications such as gas storage, molecular separation, catalysis, and drug delivery.^1–7^

An example of a widely used COF is RIO-18, a TpPa-type β-ketoenamine framework formed from 1,3,5-triformylphloroglucinol and *p*-phenylenediamine, whose structure can be seen in Figure 1. It features a theoretical pore aperture of ∼17 Å and the interlayer stacking typical of 2D COFs. Comparison of the experimental powder XRD profile of RIO-18 with the DFT-optimized theoretical pattern (Figure 3 and Section 3.1) and with reported TpPa diffractograms supports this structural assignment, while also reflecting the stacking disorder commonly observed for this family.

**Figure 1:**
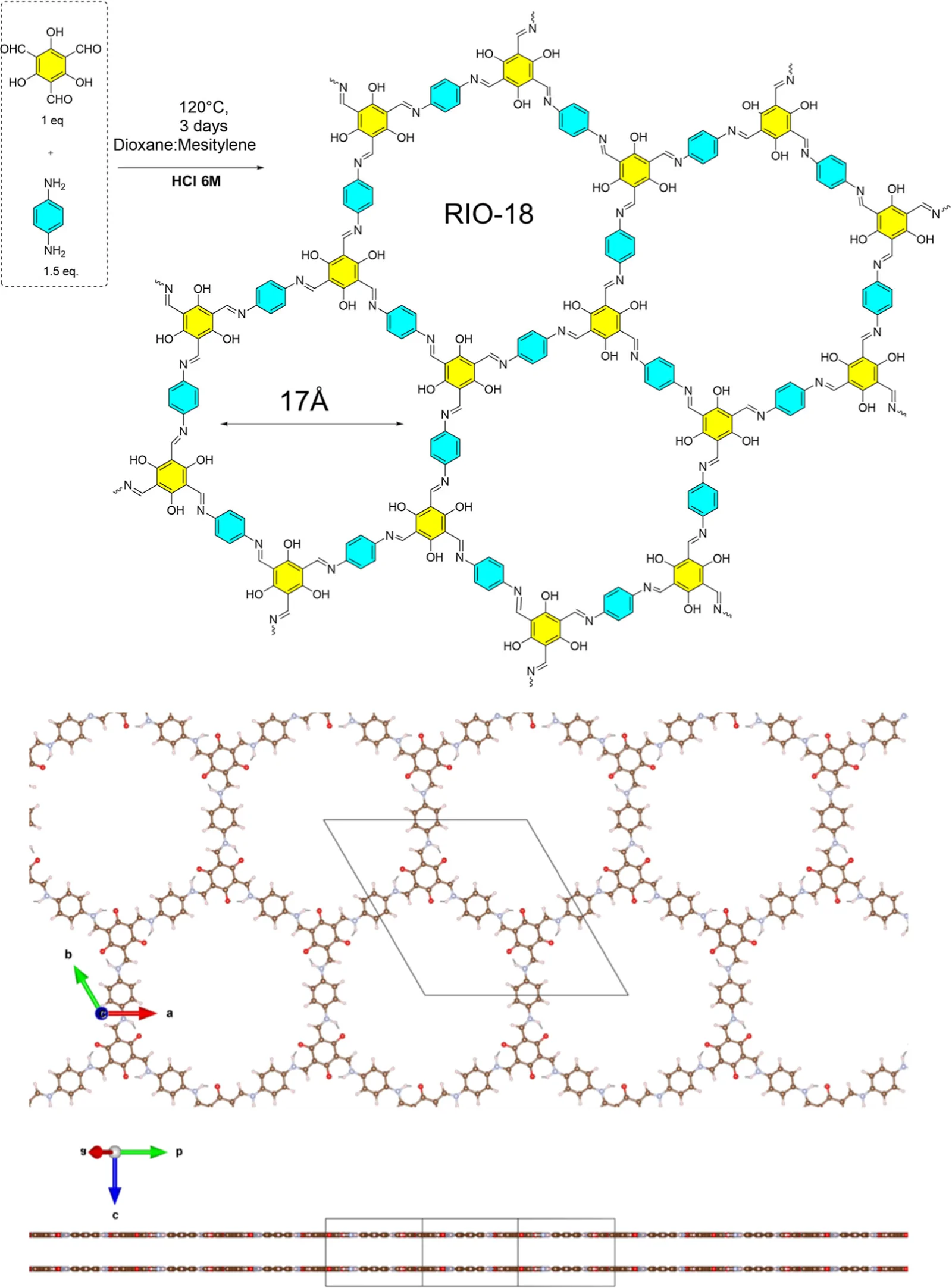
Representation of the chemical structure of COF RIO-18 indicating its synthetic parameters, theoretical pore size of 17 Å, unit cell, and interlayer space.

Despite their promising properties, the practical application of COFs is often limited by their intrinsic powder morphology, which hinders their direct use in industrial processes.^8^ To address this limitation, the incorporation of COFs into polymeric matrices has emerged as an effective strategy, leading to the development of mixed matrix membranes (MMMs).^9^ These systems combine the processability and mechanical stability of polymers with the selective adsorption and transport properties of COFs, enabling the design of materials with enhanced separation performance.^10^

Among the various applications of MMMs, gas separation—particularly CO_2_ capture—has attracted significant attention due to its environmental and industrial relevance.^11,12^ Membrane-based gas separation is an established and economically viable technology, offering advantages such as low energy consumption, operational simplicity, and scalability.^13,14^ In this context, the development of advanced membrane materials capable of improving permeability and selectivity is critical for addressing challenges associated with postcombustion gas treatment, greenhouse gas emissions and natural gas purification.^14^

Recent studies have demonstrated the potential of COF-based membranes for gas separation. For instance, COF membranes with high CO_2_ permeability and moderate selectivity, as well as hybrid MOF/COF systems, have been reported.^15,16^ In addition, computational approaches have accelerated the screening of promising materials by predicting polymer–COF interactions and transport properties.^17,18^ However, despite these advances, the relationship between COF loading, membrane morphology, and gas transport performance remains insufficiently understood, particularly in systems involving elastomeric polymers.

Table 1 reports a list of studies that address the incorporation of COFs into polymeric membranes, highlighting the reported performance and the gap relative to the present work. Direct numerical comparisons are, however, limited by differences in polymer matrix, membrane thickness, feed pressure, and test protocols.

**Table 1:** COF-Based MMMs for CO_2_ Separation Related to This work^b^T

membrane/filler	polymer matrix	performance^a^	gap relative to this work
In situ TpPa COF^19^	Pebax	P (CO_2_) ∼313 Barrer, α (CO_2_/N_2_) ∼22.6	different matrix; not a PU loading x pressure map
polymer-penetrated channels^20^	PVAm	enhanced CO_2_/N_2_, CO_2_/CH_4_ and CO_2_/H_2_ via amino channels	facilitated-transport matrix; not TpPa@PU casting MMMs
azine-linked COF^21^	matrimid	equimolar mixed-gas CO_2_/CH_4_; at 16 wt % ACOF-1, CO_2_ permeability more than doubled, with a slight increase in selectivity	different COF chemistry and polymer
COF-PBI hybrid^22^	PBI	chemically stable hybrids; pore modulation	rigid high-performance polymer, not elastomeric PU

a P (CO_2_) refers to pure gas permeability; α (CO_2_/N_2_) refers to ideal selectivity.

b Values are Those Reported by the Cited Authors Under their Stated Conditions.

RIO-18 is a suitable filler for CO_2_-selective mixed-matrix membranes because of its reported CO_2_ affinity, associated with nitrogen- and carbonyl-containing functionalities, and its well-characterized TpPa-type structure.^19^ Its pore size is substantially larger than the kinetic diameters of CO_2_ (3.3 Å), N_2_ (3.64 Å), and CH_4_ (3.8 Å),^23^ suggesting that differences in gas transport are more likely governed by gas-membrane affinity and changes in the mixed-matrix structure than by classical molecular sieving. Elastomeric polyurethane (PU) was selected as the matrix because of previously successful results^24,25^ with other fillers, as well as due to its flexibility, mechanical strength, durability, in addition to chemical and moisture resistance.^26,27^

Although some COF-based MMMs have been investigated, systematic studies addressing the combined effects of COF loading and feed pressure on the pure-gas permeation performance of elastomeric PU membranes remain limited.^21,28,29^ This work provides a systematic evaluation of RIO-18 loadings ranging from 0.1 to 5 wt % in polyurethane MMMs at feed pressures from 1.5 to 9 bar. Pure-gas permeabilities and ideal selectivities were determined for CO_2_, CH_4_, and N_2_, aiming particularly at CO_2_/N_2_ separation.

This investigation was undertaken in order to establish meaningful correlations between RIO-18 loading, operating pressure, membrane morphology, and gas transport performance. The results should provide researchers working on COF-polymer MMMs with a rationale for a reproducible membrane manufacturing process, a systematic filler loading-pressure performance map, and some evidence on the transport mechanism. The basic hypothesis here is that the separation behavior of this large-pore COF system is more consistent with differences in gas-membrane affinity and the facilitated transport provided by the filler, rather than a molecular sieving mechanism.

## 2 Materials and Methods

### 2.1 Materials

1,3,5-Triformylphloroglucinol (TP; Sigma-Aldrich, CAS 34374–88–4) and *p*-phenylenediamine (PPDA; Sigma-Aldrich, CAS 106–50–3) were used as COF building blocks. For the COF synthesis, 1,4-dioxane (Êxodo Científica, CAS 123–91–1) and mesitylene (Sigma-Aldrich, CAS 108–67–8) served as solvents, while hydrochloric acid (Isofar, CAS 7647–01–0) was employed as the reaction catalyst, without further purification. In the membrane fabrication process, tetrahydrofuran (THF; Merck, CAS 109–99–9) and ether-based polyurethane (PU; Elastollan 1185 A 10 from BASF) were used. High-purity (99.99%) CO_2_, CH_4_, and N_2_ gas standards (Linde Gases Brazil) were used for permeation tests.

### 2.2 Computational Optimization of the RIO-18 Structure

The idealized structure of RIO-18 was obtained by carrying out periodic density functional theory (DFT) calculations, using the CP2K software package (v2025.1). Electronic exchange and correlation were treated with the GGA-PBE functional, supplemented by DFT-D3 dispersions to account for long-range van der Waals interactions. A double-ζ valence polarized (DZVP) basis set was employed in conjunction with a plane-wave expansion, utilizing a cutoff energy of 600 Ry and a relative cutoff of 60 Ry. The system was modeled with a net zero charge. The initial computational model was derived by simultaneously optimizing the atomic positions and unit–cell parameters of the idealized RIO-18 structure (Figure 1, bottom). The idealized powder XRD pattern (herein referred to as “theoretical”) was simulated from the optimized geometry using the VESTA software package.

### 2.3 Synthesis of COF RIO-18

The synthesis was performed by adding the building blocks TP (2 mmol, 420 mg) and PPDA (3 mmol, 324 mg) to a glass flask (Chemglass, CG 1800 model, 48 mL), followed by the addition of 1,4-dioxane (13.6 mL) and mesitylene (1.2 mL). Subsequently, 6 mol L^–1^ hydrochloric acid (4.6 mL) was introduced. The flask was sealed and the reaction proceeded for 72 h in an oil bath at 120 °C under magnetic stirring. After this period, the product was filtered and sequentially washed with methanol (3 × 20 mL), dimethylformamide (DMF, 3 × 20 mL), and tetrahydrofuran (THF, 3 × 30 mL). The material was then soaked in THF for 72 h using a filter paper bag. Finally, the product was vacuum-dried at room temperature, yielding the COF for subsequent use and characterization.

### 2.4 Preparation of xRIO-18@PU MMM

The xRIO-18@PU membranes were prepared using the casting method. First, suspensions containing different COF loadings (0.1, 0.5, 1.0, 2.0, 3.0, and 5.0 wt % relative to PU) were prepared in THF (50 mL) by ultrasonic treatment for 5 min at high power in an ice bath to prevent solution heating. Subsequently, PU pellets (10 g) were dissolved in each suspension under magnetic stirring for 24 h. The resulting PU/RIO-18 solutions were then cast in small aliquots (5 g) onto Teflon Petri dishes, which were covered and maintained at room temperature until complete THF evaporation.

### 2.5 Characterization Methods

The COF RIO-18 and COF-containing membranes were characterized using an Ultima IV (185 mm) powder X-ray diffractometer (PXRD). Membrane Fourier transform infrared spectroscopy (FTIR) measurements were carried out using a Shimadzu spectrophotometer. Scanning electron microscopy (SEM) images were obtained using a Thermo Scientific Quattro instrument. Thermal stability of the membrane samples and of the COF was evaluated through thermogravimetric analysis (TGA) performed on a PerkinElmer Thermal Analysis system.

### 2.6 Gas Permeability Tests

The pure gas permeation properties (CO_2_, CH_4_, and N_2_) of the fabricated membranes with varying RIO-18 concentrations were evaluated, in triplicate, using a permeation system as illustrated in Figure 2. Measurements were conducted at differential pressures ranging from 1.5 to 9 bar (1.5, 3, 6, and 9 bar) and a temperature of 25 °C.

**Figure 2:**
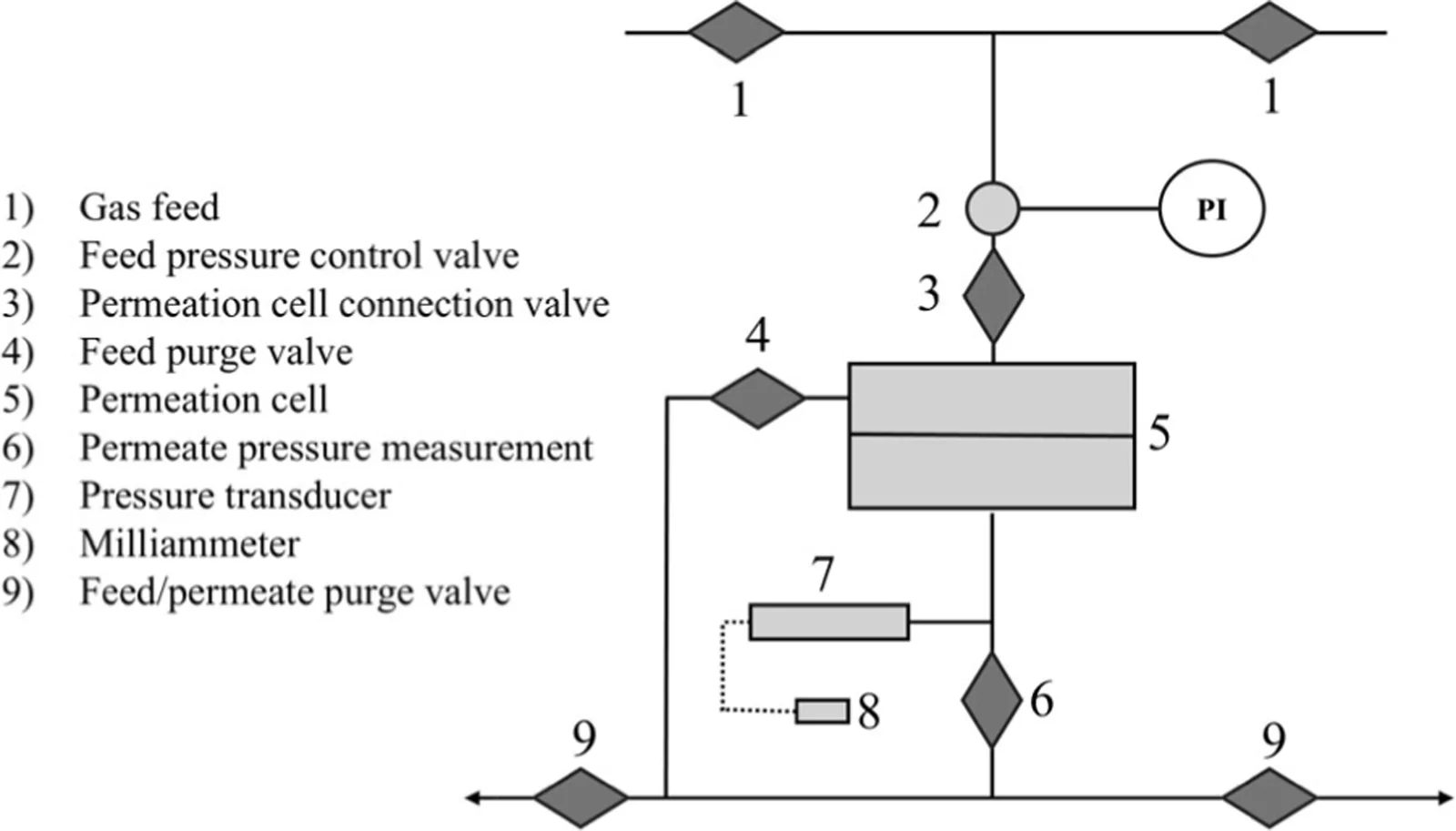
Diagram of the experimental setup of the gas permeation system.

All membranes used in gas permeation measurements had their thicknesses measured using a Mitutoyo digital micrometer, ranging from approximately 70–200 μm. All permeation experiments reported here were performed with pure gases (CO_2_, CH_4_, or N_2_). No mixed-gas permeation tests were conducted in this study; reported selectivities are therefore ideal selectivities.

The membranes were mounted in a cylindrical test cell, yielding an effective membrane area of 8.5 cm^2^. Pressure variation on the permeate side was recorded using LogChart II software until a linear behavior was established. Gas permeability P_i_ values were calculated using eq I.^13,30^

I $$P_{i}=\left(\frac{Q_{i}l}{A\Delta p}\right)$$

The permeability for each gas *i* was expressed in Barrer (1 Barrer = 1 × 10^–10^ cm^3^ (STP) cm (cm^2^s cmHg)^−1^). In this equation, *Q*_*i*_ represents the flow rate of the analyzed gas, *l* is the membrane thickness, *A* represents the effective membrane area, and Δ*p* is the pressure difference between the feed and permeate sides.

The ideal selectivity of a gas A with respect to a gas B, α_A/B,_ was calculated from eq II.^13,30^

II $$\alpha_{A/B}=\frac{P_{A}}{P_{B}}$$

## 3 Results and Discussion

### 3.1 Characterization of COF RIO-18

For the characterization of RIO-18, powder X-ray diffraction was performed. This technique is used to verify the successful synthesis of the COF, based on the comparison between the experimental and theoretical crystallinity data, obtained through computational calculations.

Figure 3 compares the experimental X-ray diffraction pattern of RIO-18 with its theoretical counterpart. The results confirm the formation of a crystalline material, displaying characteristic COF reflections. The experimental profile shows a high-intensity peak near 4.7° and a broad feature around 27°, which are commonly associated with in-plane periodicity and interlayer (001) stacking in 2D beta-ketoenamine COFs such as TpPa.^31,32^ The small deviation in peak positions between the experimental and simulated PXRD patterns (Figure 3), especially in the region of 20–30°, is likely due to difficulties for DFT to predict the interlamellar *d*-spacing correctly. This is a known nuance of DFT modeling when accounting for weak interlayer π–π stacking interactions, which slightly overestimates the stacking tightness compared to experimental observations. The predicted DFT interlayer spacing is 3.97 Å, while the experimental data indicates that the interlayer distance is about 3.3 Å (peak at 27°, similar to the one in graphite). DFT accurately computes the dimensions of the *a* and *b* axis, since it properly describes chemical bonding in those materials, which defines these crystallographic axes, leading to better agreement in the lower 2θ region of the diffractogram. In addition, the several reflections between approximately 22° and 25°, poorly resolved experimentally compared with the simulated pattern, are consistent with stacking disorder/imperfect interlayer registry often reported for TpPa-type COFs and may also reflect residual framework strain and stacking faults. Therefore, while the intense low-angle peak and the ∼27° feature support a crystalline 2D COF architecture, a more accurate description is in-plane crystalline order with imperfect interlayer stacking, rather than fully ideal long-range three-dimensional order. The overall agreement between experimental and theoretical profiles validates the successful synthesis of RIO-18.

**Figure 3:**
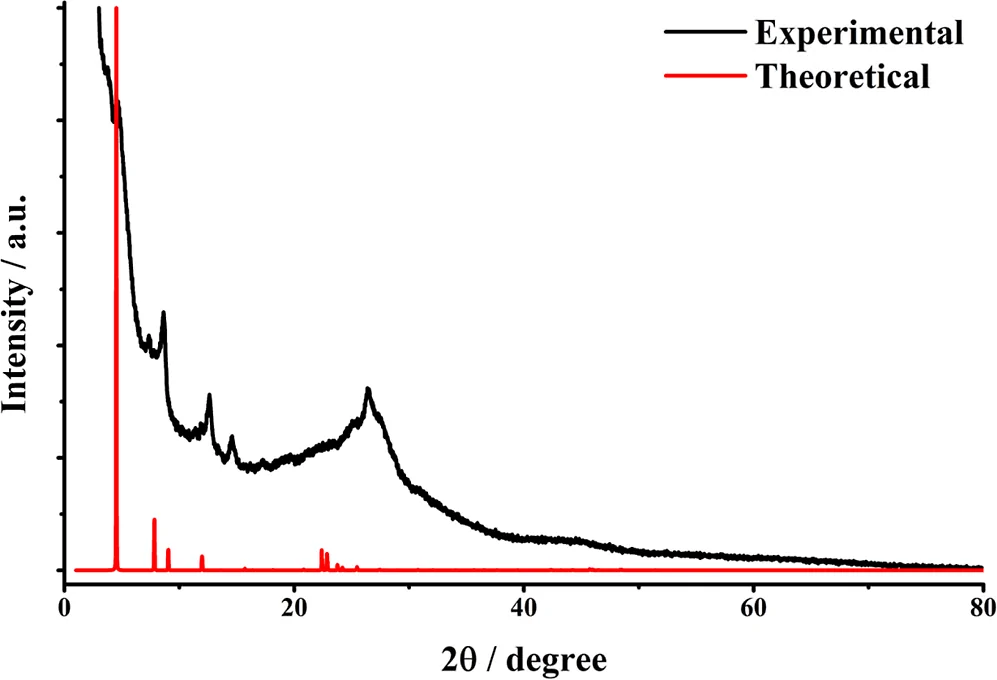
Normalized experimental and theoretical powder X-ray diffractogram of RIO-18.

### 3.2 Characterization of xRIO-18@PU MMM

Considering that this study focuses on the impacts of incorporating RIO-18 into polyurethane membranes, a series of characterization techniques were employed to evaluate this effect, including chemical, thermal, and morphological analyses. Each of these techniques aims to elucidate the stability of the membranes, their chemical and structural integrity, and to provide a better understanding of the gas diffusion process during permeation.

The physical aspects of the casting suspension can be observed in Figure 4a. The uniformity of the suspension is evident, as well as the absence of visible solid agglomerates in the THF medium, which acts not only as a stabilizer for the suspended COF particles but also as a solvent for the complete dissolution of polyurethane. The choice of solvent at this stage is crucial in the synthesis process, since its evaporation rate can influence the membrane morphology; for example, the formation of bubbles may cause the membrane to transition from a dense to a porous structure.

**Figure 4:**
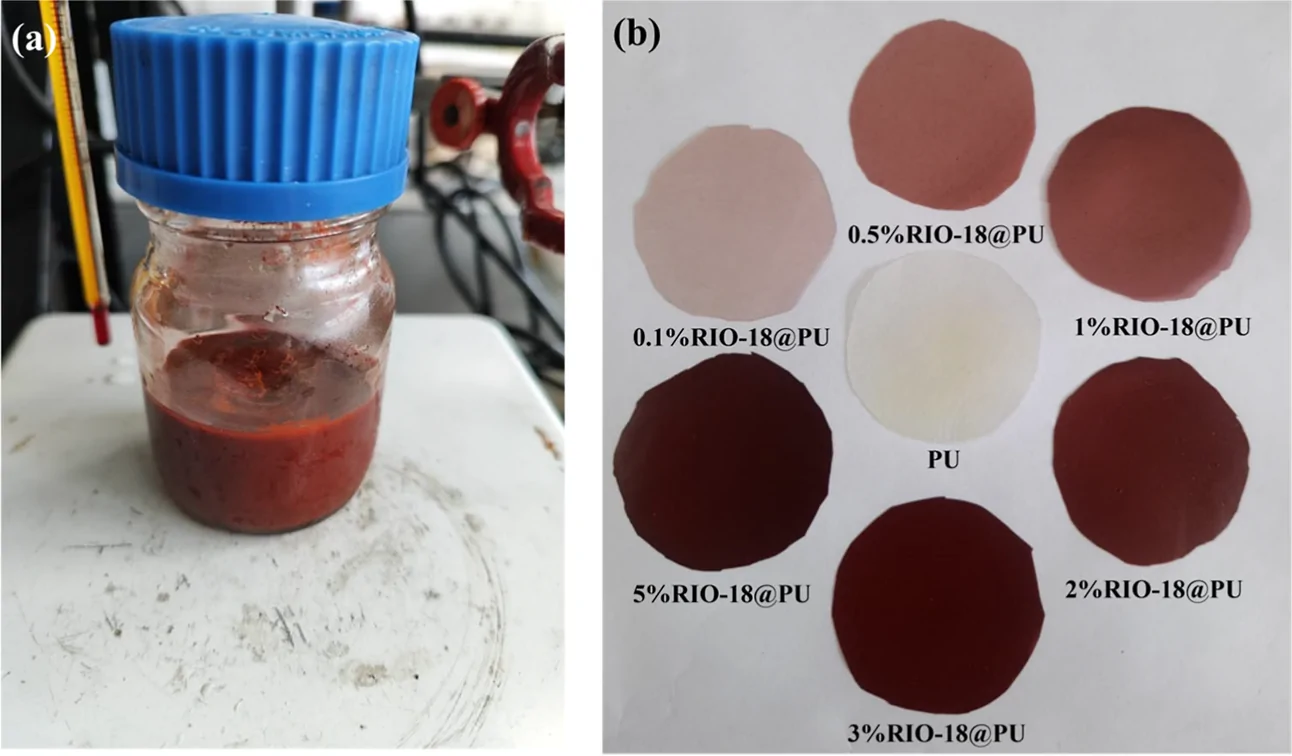
(a) Digital photograph of the casting suspension of 1% RIO-18@PU and (b) digital photos of cut pieces of PU, 0.1% RIO-18@PU, 0.5% RIO-18@PU, 1% RIO-18@PU, 2% RIO-18@PU, 3% RIO-18@PU and 5% RIO-18@PU MMM.

The slow evaporation of the solvent allowed the formation of a dense and uniform RIO-18 membrane within the polyurethane matrix.^33^ This uniformity can be observed in Figure 4b. It is noted that even small variations in the COF loading result in clear visual changes in the membrane coloration. This can be explained by the low density of the material, which leads to a relatively large volume of powder that must be dispersed during membrane preparation for a given weight fraction. The obtained membranes were easily detached from the Teflon Petri dish and stored without any further treatment prior to the gas permeation tests.

It was observed during the preparation of the membranes that, at a concentration of 5% RIO-18, the membrane exhibited a coloration similar to that of the pure COF. In addition, the amount of solid suspended in THF and preliminary permeation results suggested that this concentration range could be considered the upper limit in the concentration sweep investigated. Similarly, at a concentration of 0.1% RIO-18, in addition to the slight coloration in the nearly colorless membrane, the limitations associated with weighing and suspension preparation at low COF mass quantities served to define this concentration as the lower limit used in the study.

The X-ray diffractograms of the membranes (Figure 5) show that the sample retains the characteristics of the PU matrix, when compared to the pristine COF, although traces of crystallinity are still observed in some samples, such as 2% RIO-18@PU. This probably originates from COF particles on the surface of the analyzed membrane. The low crystallinity of the membranes is expected, since most of their composition still consists of elastomeric polyurethane.^34^

**Figure 5:**
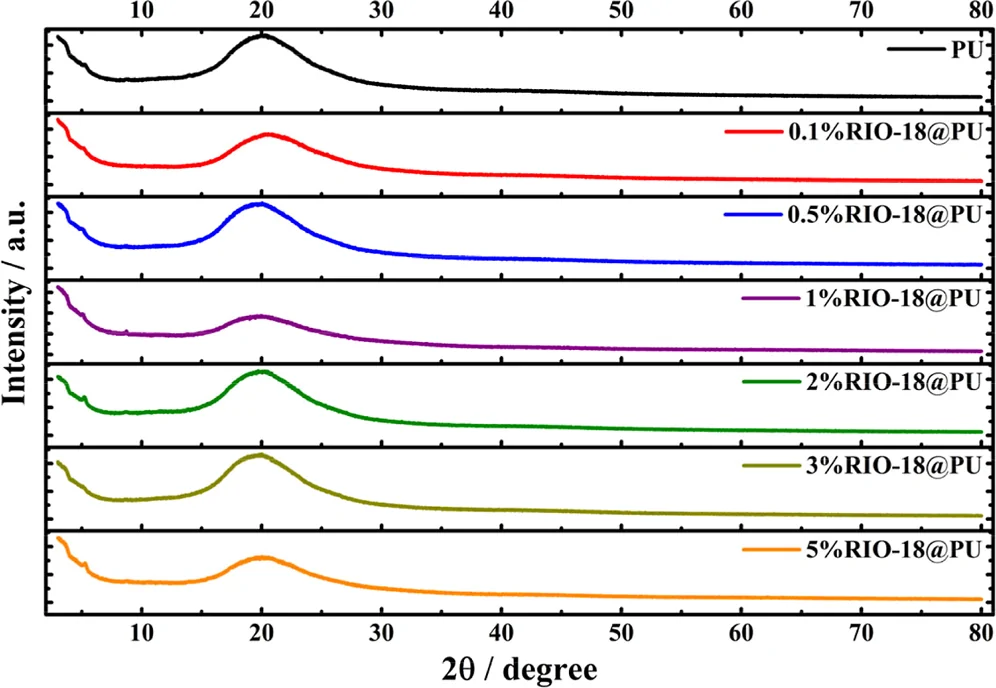
X-ray diffractograms of 0.1% RIO-18@PU, 0.5% RIO-18@PU, 1% RIO-18@PU, 2% RIO-18@PU, 3% RIO-18@PU and 5% RIO-18@PU MMMs compared to the pure PU membrane.

In addition to the predominantly amorphous nature of the membrane, the diffraction profiles do not show significant variations with different RIO-18 loadings, indicating that the incorporation of the COF did not result in relevant structural changes in the PU matrix. Unlike COFs, the low crystallinity of the polymer is not a predominant factor, since the diffusion of species through the membrane depends rather on other properties such as their chemical nature, the intermolecular spacing, and operational parameters such as temperature and pressure.

The infrared spectra of the membranes with different COF concentrations were compared with that of the pure polymer to evaluate possible chemical modifications induced by the incorporation of the COF (Figure 6). In all analyzed membranes, the characteristic peaks of polyurethane are observed overlapping those of RIO-18. The C=O stretching band is noted around 1700 cm^–1^, the C–O stretching near 1100 cm^–1^, the N–H band around 3300 cm^–1^, and the C–N stretching near 1600 cm^–1^.

**Figure 6:**
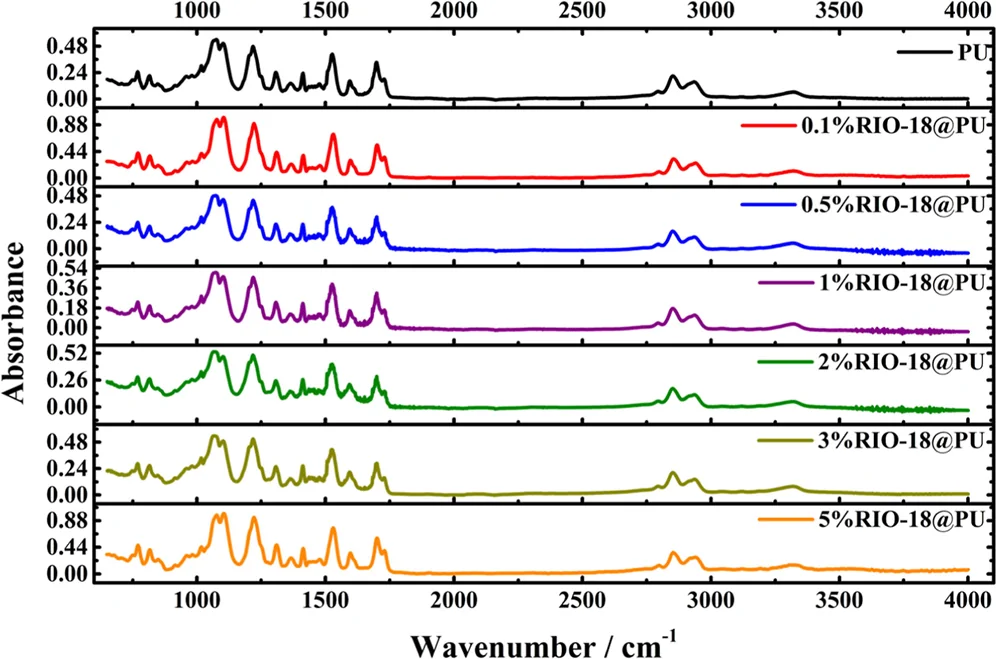
FTIR spectra of COF RIO-18 MMMs compared to the pure PU membrane. Absorbances are normalized.

Since the COF is distributed throughout the membrane and its relative small concentration ranges from 0.1 to 5 wt % relative to the polymer, it should be expected that the spectra would exhibit a profile predominantly similar to that of the unloaded polymer. These results indicate that the incorporation of RIO-18 into PU did not promote detectable chemical changes in the polymer matrix. Bands associated with C=O and N–H functionalities—known CO_2_-philic moieties in TpPa-type COFs^19^—remain available in the composite, although their contribution to transport is inferred from permeation trends and literature rather than from direct sorption measurements in this work.

The SEM micrographs presented in Figure 7 allow a better understanding of the dispersion of RIO-18 throughout the membrane, both on its surface and within its interior. The observed results corroborate the hypothesis derived from the different colorations of the membranes regarding how RIO-18 is distributed throughout the membrane. In addition, details concerning defects, orientation, and the distribution pattern of the particles can be obtained.

**Figure 7:**
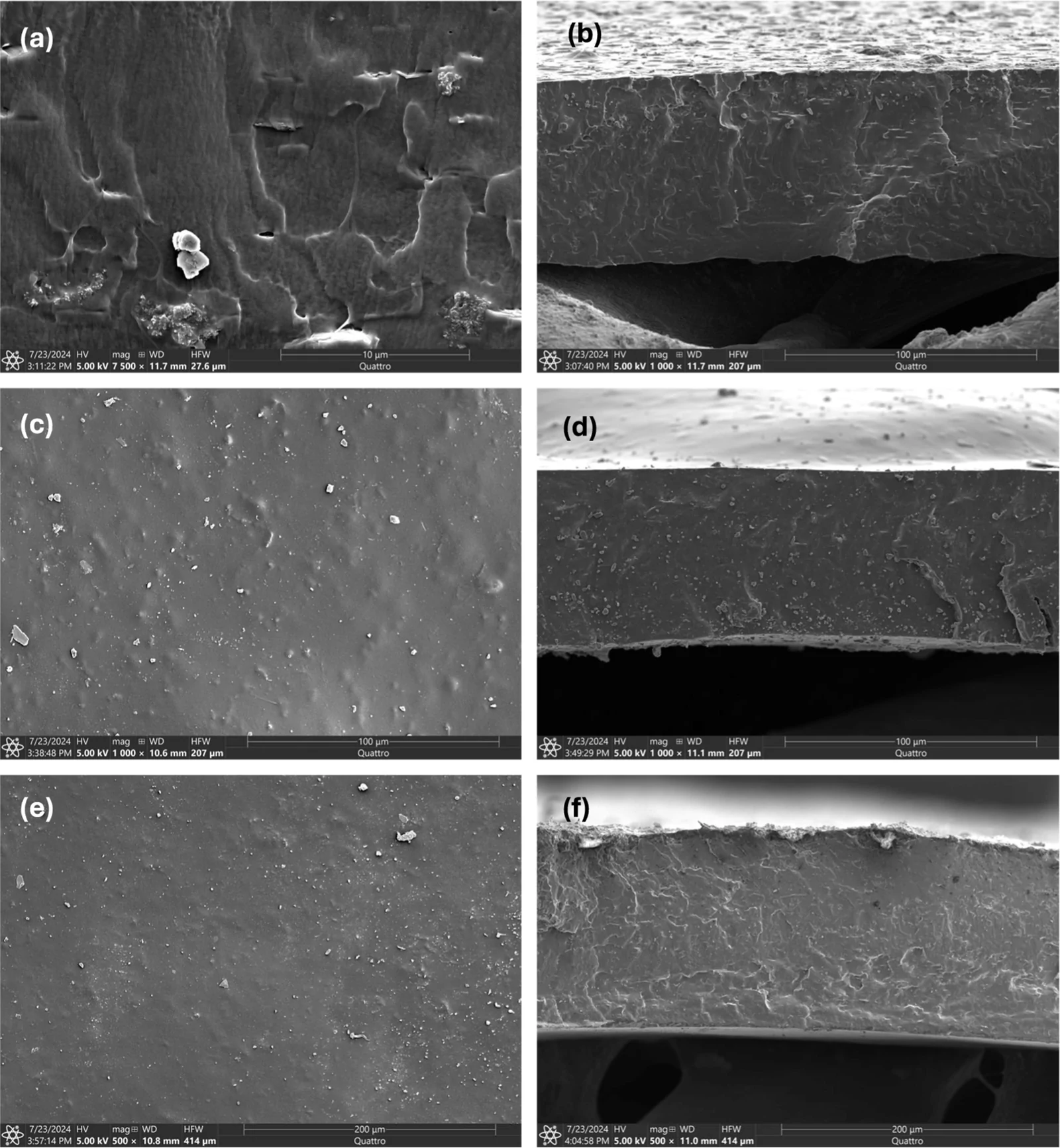
Scanning electron microscopy photomicrographs of the surface and cross section of the (a,b) 2% RIO-18@PU, (c,d) 3% RIO-18@PU and (e,f) 5% RIO-18@PU MMM.

In the analyzed SEM regions, no major defects or vacancies typically associated with uncontrolled solvent evaporation were observed. This supports the formation of dense membranes suitable for permeation testing, although SEM cannot exclude the presence of sparse defects outside the imaged areas. Some larger RIO-18 agglomerates, which were not visible in the suspension, can be observed; nevertheless, these agglomerates remain dispersed within the observed membrane samples. The presence of these agglomerates helps explain why the highest RIO-18 concentration used in this work did not exceed 5%, since higher filler loads could lead to the formation of even larger aggregates that may affect the structural quality of the membrane and the separation process. This observation is consistent with the uniform coloration observed in the membranes. The cross-sectional images show that the membrane was formed without the presence of bubbles or related defects (Figure 7d). It is also observed that the RIO-18 particles are well distributed along the direction of solvent evaporation, indicating that no evidence of sedimentation of the COF was observed at the bottom of the MMM during the process.

The uniform distribution of RIO-18 across both the surface and the interior of the membrane is fundamental to ensuring the reliability of the results obtained during the gas permeation stage. The absence of significant defects and the lack of observable bubbles in the analyzed regions suggest that the membranes were successfully prepared for permeation testing, although local agglomerates were observed at higher RIO-18 loadings, as discussed later.

The TGA results serve as a basis for evaluating the stability of the MMMs during the separation process, considering their thermal robustness under typical laboratory separation conditions, and also provide information on how the incorporation of RIO-18 influences the properties and thermal degradation of the polymeric membrane.

Figure 8 shows a comparison between the thermogravimetric analyses of the pure polymer and the 5% RIO-18@PU membrane. From this comparison, it is possible to observe the similarity in the thermal behavior of the materials.

**Figure 8:**
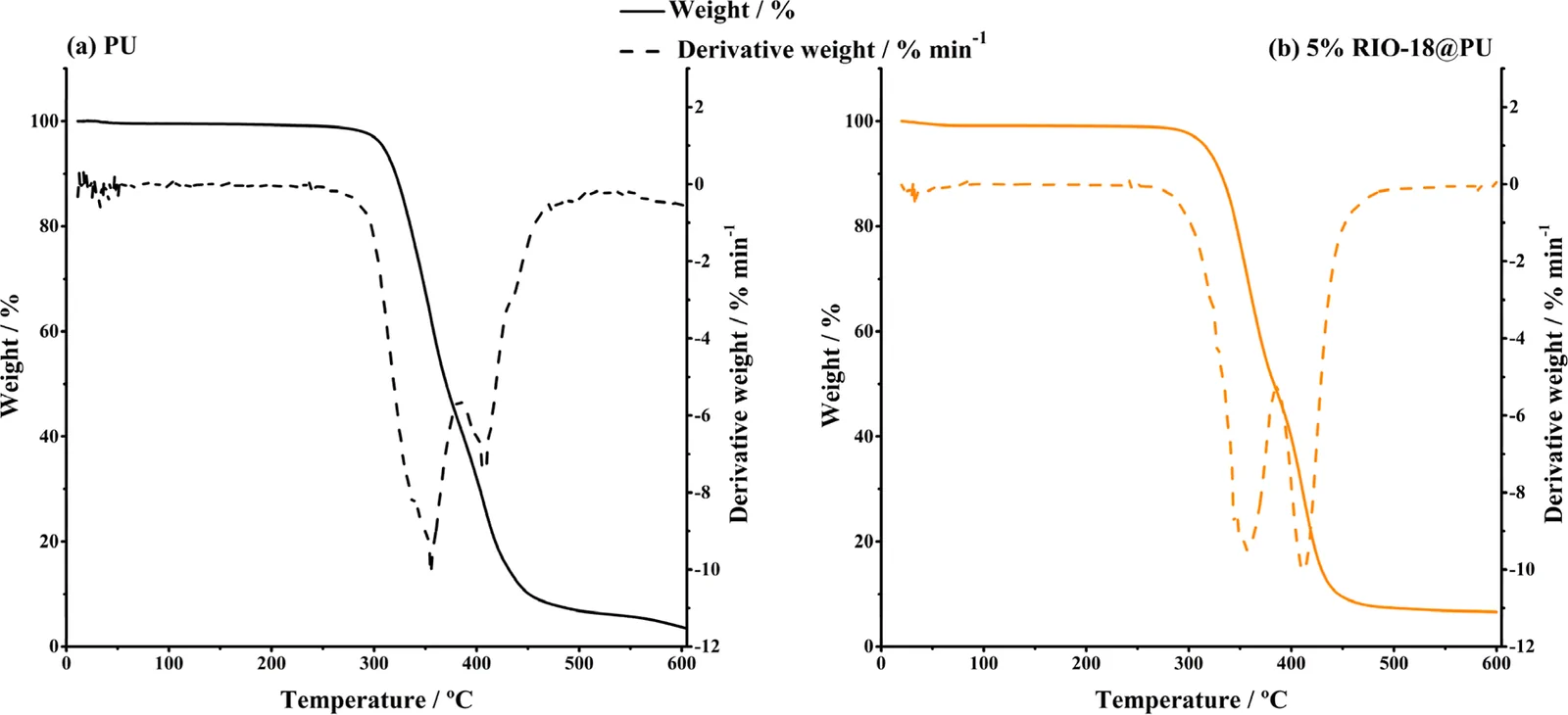
Thermogravimetric analysis of (a) PU and (b) 5% RIO-18@PU membrane.

In both samples, a slight mass loss is observed up to 100 °C, attributed to the loss of residual moisture and volatile species present in the samples. Since this mass loss is very small, it can be inferred that the moisture content in the membranes is low, which is important because water molecules can occupy the adsorption sites of the COF and hinder the gas diffusion process.^35^

The major mass loss occurs between 350 and 450 °C, where the material undergoes almost complete degradation. This degradation corresponds to the thermal decomposition of the polyurethane backbone and urethane linkages. This behavior is associated with the fact that the material is predominantly composed of light elements linked by covalent bonds (carbon, nitrogen, oxygen, and hydrogen). After 450 °C, a slow decrease in the mass percentage is observed, which, in this temperature range, corresponds to the residual products from membrane degradation.

Furthermore, microscopy indicates that RIO-18 particles are generally well distributed in the analyzed regions, with no major defects observed in those areas, supporting the reliability of the subsequent pure-gas permeation measurements. The compatibility between RIO-18 and the polyurethane matrix, both organic materials sharing similar functional groups (imine, carbonyl, and aromatic moieties), favors interfacial interactions that contribute to the thermal stability of the MMM. This stability is an important outcome in the study of the influence of the COF on the polymeric matrix. Once the questions regarding the chemical and structural feasibility of incorporating RIO-18 into PU membranes were clarified, the gas permeation stage could be initiated.

### 3.3 Gas Permeability of RIO-18 COF/PU MMM

Gas permeation experiments were undertaken to check the effect of COF content and operating pressure on both the permeability of the target gases and their ideal selectivity.

Figure 9a shows the CO_2_ permeation results at a pressure of 3 bar for each of the membranes investigated. Meanwhile, Figure 9b presents the CO_2_ permeation results for each membrane at all the pressures explored in this investigation. These results served as a screening to define the most suitable operating range of the membrane, as well as to identify a behavioral pattern resulting from the incorporation of the COF into the membrane.

**Figure 9:**
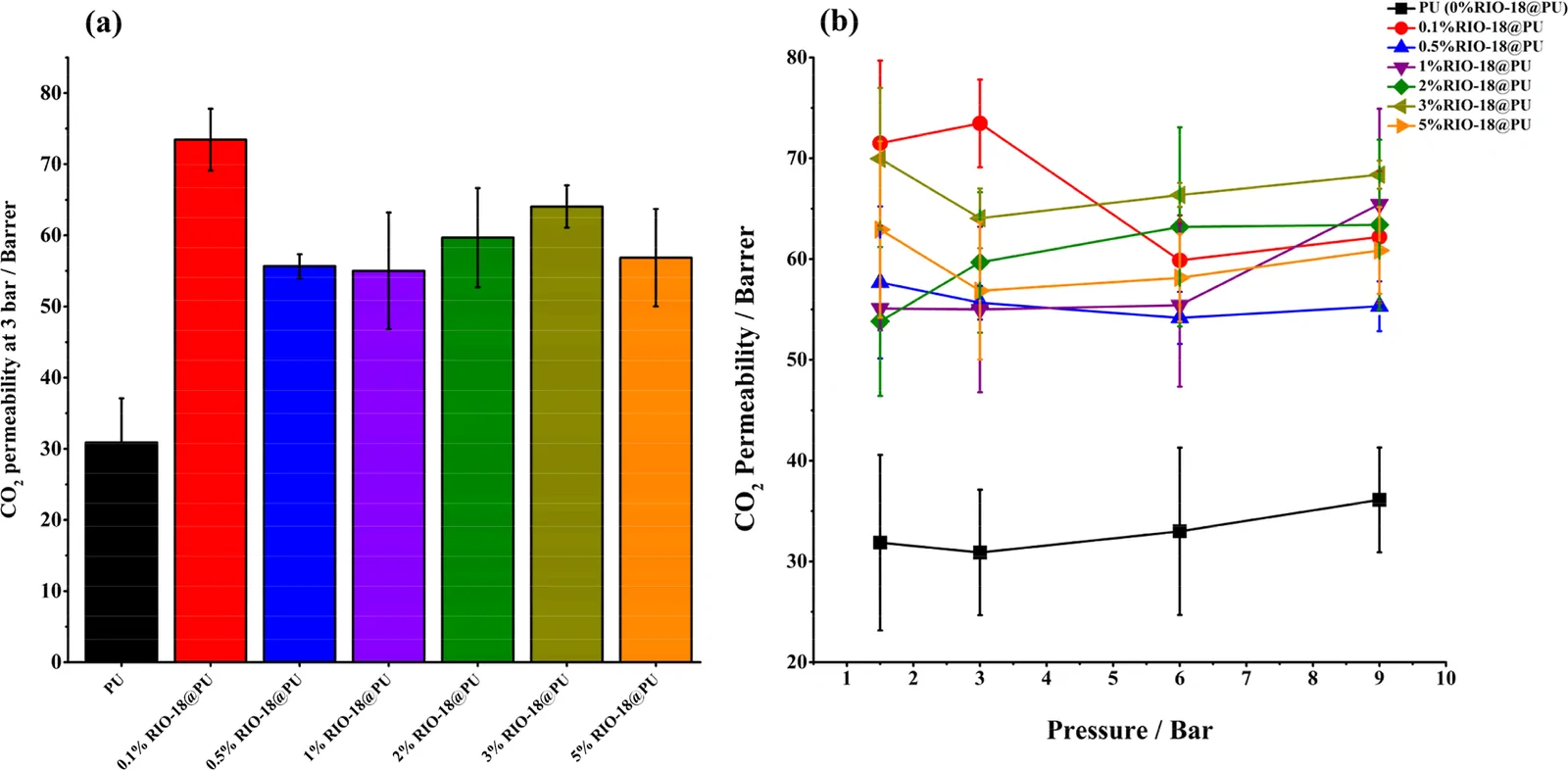
(a) CO_2_ permeability at 3 bar for all *x*RIO-18@PU and (b) effect of RIO-18 content and pressure on CO_2_ permeability at 1.5, 3, 6, and 9 bar for all *x*RIO-18@PU. Error bars represent the standard deviation of triplicate measurements.

As shown in Figure 9, the incorporation of the RIO-18 COF into PU MMMs resulted in a general increase in CO_2_ permeability compared to the pure PU membrane, observed at all COF loadings over the pressure range studied (1.5–9 bar). A substantial increase of 138% is observed for the 0.1% RIO-18@PU membrane at 3 bar (73.47 Barrer) compared to PU under the same conditions. The lowest permeability value was observed for the 2% RIO-18@PU membrane, with 53.82 Barrer, corresponding to an increase of 69% compared to PU at 1.5 bar.

For a clearer visualization of the influence of concentration on CO_2_ permeation through the membrane, Figure 10 presents a contour map of CO_2_ permeability as a function of RIO-18 loading and feed pressure. From this graph, it is possible to observe a clear nonlinear relationship between permeation and concentration, with the highest values occurring at very low and intermediate concentrations. These results suggest that multiple factors influence the permeation process beyond the variation in RIO-18 loading.

**Figure 10:**
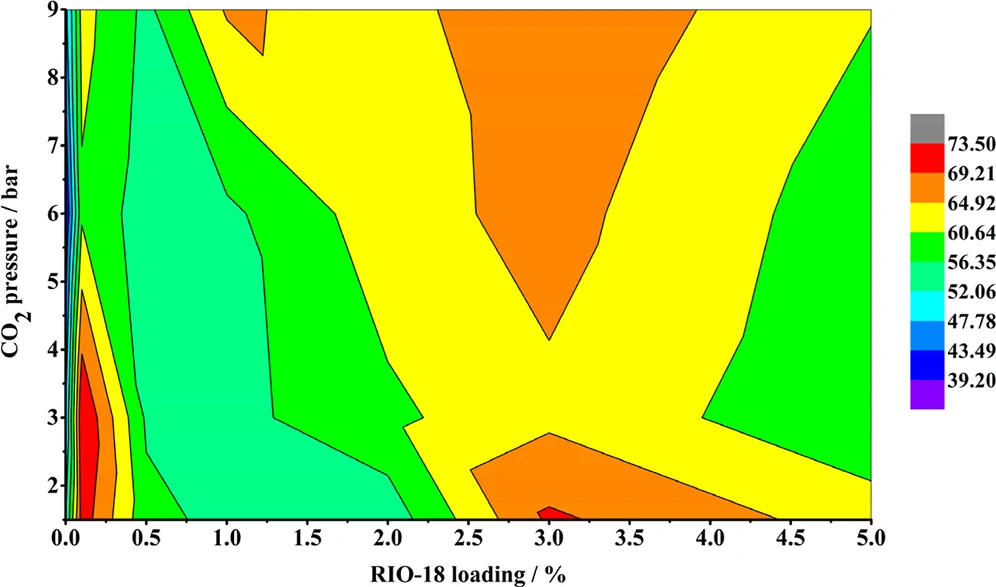
Contour map of CO_2_ permeability as a function of RIO-18 loading and feed pressure for *x*RIO-18@PU MMMs.

Although computational simulation of the gas diffusion process in membranes can be challenging, since the tools required to accurately reproduce experimental conditions are costly and complex, it may be helpful to elucidate the gas transport mechanisms in these membranes. There is evidence that the pores may contribute to enhancing molecular transport through the membrane, thereby improving the efficiency of the process.^36^

The increase in CO_2_ permeability in RIO-18@PU MMMs is consistent with preferential interactions between CO_2_ and CO_2_-philic sites of the RIO-18 framework (C=O and N–H), as discussed for related COFs,^37^ together with additional diffusion pathways associated with dispersed filler particles. In a solution-diffusion framework, permeability is the product of solubility and diffusivity (*P* = *S* × *D*); however, solubility coefficients, diffusivity values, and sorption isotherms were not measured in this study. Consequently, the adsorption-assisted contribution is suggested as a physically plausible hypothesis supported by the larger relative enhancement for CO_2_ versus N_2_, the chemical nature of RIO-18, and previous literature results on TpPa-type fillers.

On the other hand, higher COF concentrations lead to a reduction in CO_2_ permeability. This effect can be attributed to the presence of small COF agglomerates observed by SEM, which create barriers to gas diffusion (tortuosity effect).^19^ This result suggests that there is a 2-fold pathway in which, while the COF can enhance permeability through adsorption-assisted transport via adsorption sites, a relative excess of COF during the membrane fabrication process may lead to the formation of agglomerates, reaching a limit beyond which the diffusion of CO_2_ through the membrane is hindered, resulting in a loss of transport efficiency.

Regarding the effect of pressure, higher permeability values are observed at lower pressures, followed by a reduction and a subsequent increase as the pressure rises. This may reflect a stronger relative contribution of CO_2_ sorption effects at lower pressures, where COF sites may play a larger role, whereas at higher pressures transport becomes increasingly governed by solution–diffusion through the mixed-matrix structure. In this case, the increase in pressure helps CO_2_ molecules to diffuse through the membrane in such a way that the adsorption sites are less effective.

Therefore, the RIO-18 loading, gas pressure, and the barrier effect caused by COF agglomerates appear to govern the permeation process in xRIO-18@PU MMMs, suggesting that these properties can be manipulated to optimize the permeation performance of these membranes.

The selectivity behavior also allows some observations on the role of COF RIO-18. Table 2 presents the ideal selectivity for each of the concentrations studied at a pressure of 3 bar.

**Table 2:** CO_2_/CH_4_, CO_2_/N_2_ and CH_4_/N_2_ Ideal Selectivity (α) for All *x*RIO-18@PU at 3 bar

membrane	α CO_2_/CH_4_	α CO_2_/N_2_	α CH_4_/N_2_
PU	11.0	14.4	1.3
0.1% RIO-18@PU	14.6	45.0	3.1
0.5% RIO-18@PU	11.4	54.1	4.7
1% RIO-18@PU	11.1	32.9	3.0
2% RIO-18@PU	12.1	43.6	3.6
3% RIO-18@PU	14.8	79.8	5.4
5% RIO-18@PU	12.4	37.3	3.0

The CO_2_/N_2_ ideal selectivity was markedly enhanced with the incorporation of the COF, reaching 79.8 for the membrane containing 3% RIO-18, representing a 454% increase compared to pure PU. Furthermore, the CO_2_/CH_4_ ideal selectivity remained unchanged or slightly higher when compared to PU, up to a 34% increase. This behavior can be attributed to a slight increase in CH_4_ permeability along with CO_2_, while N_2_ permeability did not show a significant increase and even exhibited a slight decrease.

The nonmonotonic dependence of CO_2_ permeability on RIO-18 loading (Figures 9 and 10 and Table 2) deserves explicit comment. The highest CO_2_ permeability is observed at the lowest filler content (0.1 wt %), whereas intermediate loadings (notably ∼2 wt %) yield comparatively lower permeabilities before performance partially recovers at other concentrations; ideal CO_2_/N_2_ selectivity reaches its maximum at 3 wt % (α = 79.8). This is consistent with competing morphological effects: (i) at very low loading, particles are more likely well isolated/wetted by PU, maximizing additional pathways with minimal tortuosity; (ii) at intermediate-higher loadings, localized agglomerates increase tortuosity; (iii) selectivity does not peak at the same loading as permeability. Thus, 0.1 wt % maximizes CO_2_ permeability, whereas 3 wt % maximizes ideal CO_2_/N_2_ selectivity under Table 2 conditions.

It is also worth noting that facilitated transport in filler-containing polymer membranes is often more evident at lower feed pressures and typically becomes less pronounced as pressure increases, owing to saturation of the interactive sites. This pressure-dependent contribution may therefore help explain the higher CO_2_ permeabilities observed under milder feed conditions, in addition to the morphological effects of RIO-18 loading discussed above.

Taken together, the large enhancement of ideal CO_2_/N_2_ selectivity with only modest changes in CO_2_/CH_4_ selectivity indicates that the primary application niche of *x*RIO-18@PU MMMs is CO_2_/N_2_ separation, as encountered in postcombustion flue-gas contexts. Claims related to direct air capture (DAC) would require substantially higher permeances (thinner active layers) and dedicated process analysis beyond the scope of this work. As previously mentioned, the kinetic diameters of the studied gases (3.3 Å for CO_2_, 3.64 Å for N_2_, and 3.8 Å for CH_4_) are approximately five times smaller than the average pore size of the COF (17 Å). The similarity in the molecular sizes of these species makes classical molecular sieving within the COF pores unlikely, in which one species would be blocked in favor of another, although the COF may provide preferential diffusion pathways for gas transport through the membrane.

Since the pore diameter of the COF is considerably larger than the kinetic diameters of the target gases, steric hindrance effects are expected to be minimal, indicating that the differences in permeation are more closely related to gas diffusivity and affinity with the membrane structure than to molecular sieving effects. Even though larger pores can favor Knudsen-type diffusion mechanisms, in which gas transport is influenced by molecular mass rather than molecular size, the membrane behaves as a dense mixed-matrix system, suggesting that the transport mechanism remains predominantly associated with the solution-diffusion model.^30^

Although the COF may favor CO_2_ permeation through preferential adsorption sites, the other species may also benefit from this effect, albeit to a lesser extent. CO_2_ exhibits high condensability and solubility in polymeric membranes, favoring stronger interactions with both the polyurethane matrix and the adsorption sites present in the COF structure. The presence of the COF may further contribute to CO_2_ transport through the membrane by increasing local sorption and providing preferential diffusion regions.

On the other hand, CH_4_ appears to benefit mainly from the additional free volume provided by RIO-18 within the membrane, which slightly favors its diffusion through the polymeric matrix; however, its low affinity for the membrane structure still limits its permeation compared to CO_2_. N_2_, in turn, permeates the polymeric membrane to a much lesser extent due to its comparatively low condensability and low affinity for both the polymeric matrix and the COF structure, and the addition of the COF was not sufficient to significantly facilitate its diffusion through the membrane. These factors are consistent with the observed changes in the ideal selectivity of the studied membranes. Within the studied pressure range, no evidence of pressure-induced molecular sieving effects was observed, reinforcing that the separation behavior is predominantly associated with differences in gas diffusion and sorption within the mixed-matrix structure.

Only ideal selectivities are reported, as gas mixture permeation were not performed. Competitive sorption, plasticization, and concentration polarization can reduce real selectivities relative to ideal values; therefore, the ideal selectivities should be regarded as screening metrics rather than direct predictors of real systems performance. Comparing the results of the *x*RIO-18@PU membranes with literature data (Figure 11), it is observed that, although they do not surpass the 2008 Robeson upper bound, they approach this limit in the CO_2_/N_2_ system. These results are encouraging for proof-of-concept CO_2_/N_2_ separation materials based on RIO-18/PU MMMs.

**Figure 11:**
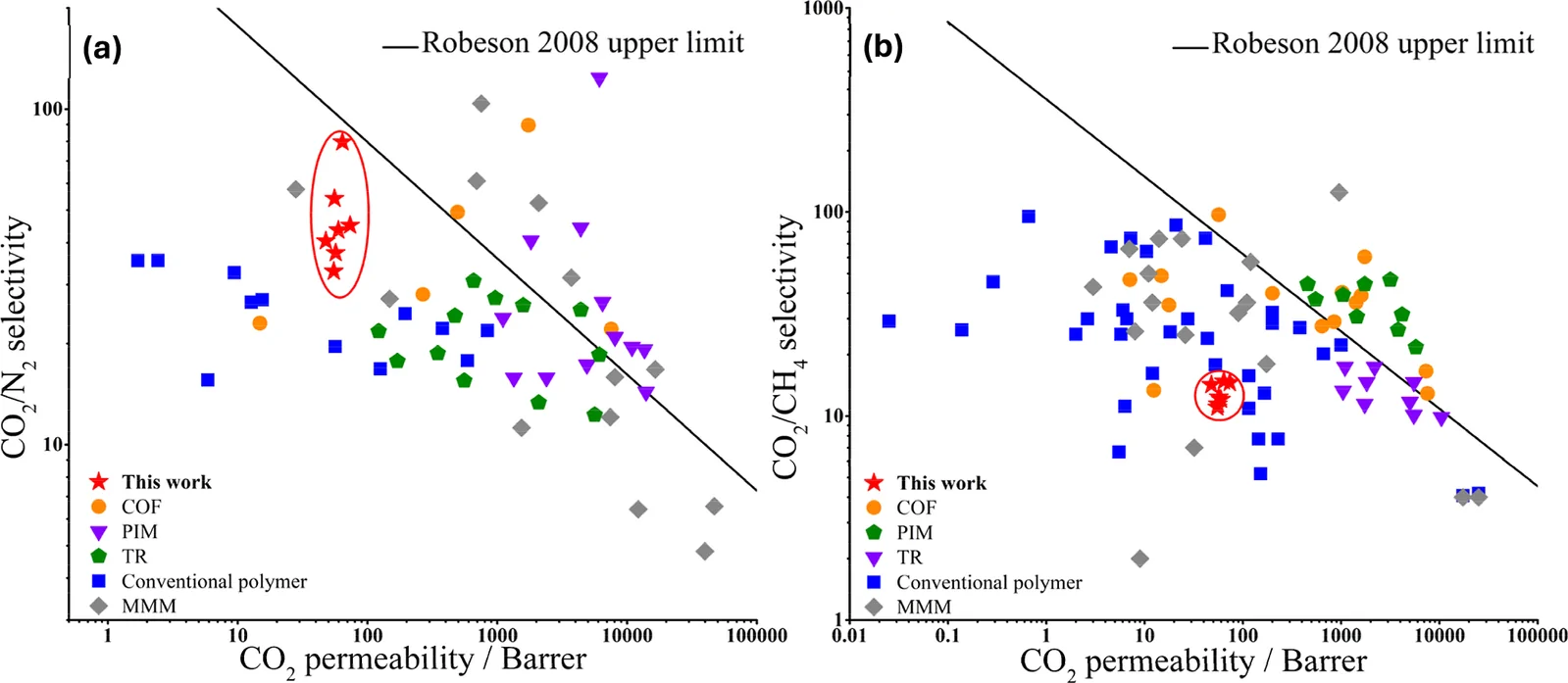
Performance of xRIO-18@PU MMM: ideal selectivity and CO_2_ permeability for (a) CO_2_/N_2_ and (b) CO_2_/CH_4._ ^20,38–41^ Literature data points and the 2008 Robeson upper bound were replotted by the authors from the cited sources for comparison; the figure was prepared by the authors.

Noteworthy, the membranes prepared here are relatively thick (70–200 μm), and all transport data are pure-gas measurements at 25 °C. We did not evaluate gas mixtures, as well as CO_2_-induced plasticization under prolonged high-CO_2_ exposure, physical aging, humidity effects, or long-term mechanical/chemical stability—all of which may be critical for industrial membrane module operation. Comparison with commercial gas-separation membranes should therefore remain qualitative: commercial membranes typically are thin-film composites with much higher permeance and are qualified to operate under process-specific conditions. The contribution of the present work is demonstrating that low-to-moderate RIO-18 loadings can markedly improve ideal CO_2_/N_2_ selectivity of PU, motivating future thinner composite layer membranes.

Further optimization, in particular reducing the selective-layer thickness via thin-film composite architectures, will be required before any assessment of process-scale applicability. The present freestanding films should be viewed as model MMMs that establish composition-performance relationships for RIO-18 in PU.

## 4 Conclusion

Mixed-matrix membranes based on polyurethane containing different loadings of COF RIO-18 (TpPa-type) were successfully prepared and evaluated by pure-gas permeation. Incorporation of RIO-18 led to a maximum CO_2_ permeability of ∼73 Barrer (138% increase vs pure PU at 3 bar for 0.1%RIO-18@PU). The ideal CO_2_/N_2_ selectivity increased by up to 454% (α = 79.8 at 3% RIO-18@PU), whereas CO_2_/CH_4_ selectivity increased by up to ∼34%. Performance is nonmonotonic with loading: the lowest filler content maximized CO_2_ permeability, while 3 wt % maximized ideal CO_2_/N_2_ selectivity, consistent with a balance between beneficial filler—matrix pathways and agglomeration-induced tortuosity at higher loadings. Within the limits of the available characterization, RIO-18 is generally well distributed in the analyzed membrane regions, and the permeation trends are consistent with preferential CO_2_ transport relative to N_2_. Because sorption isotherms and diffusivity were not measured, competition between solubility and diffusivity enhancements remains open. The results point primarily to CO_2_/N_2_ separation (postcombustion–relevant pairs) as the main application, while more engineering developments are needed for appropriate evaluations.

## References

[1] Li X., Kawai K., Fujitsuka M., Osakada Y. (2021). COF-Based Photocatalyst
for Energy and Environment Applications. Surf.
Interfaces.

[2] Ding S. Y., Wang W. (2013). Covalent Organic Frameworks
(COFs): From Design to Applications. Chem. Soc.
Rev..

[3] Zhang K., Kirlikovali K. O., Varma R. S., Jin Z., Jang H. W., Farha O. K., Shokouhimehr M. (2020). Covalent Organic Frameworks: Emerging
Organic Solid Materials for Energy and Electrochemical Applications. ACS Appl. Mater. Interfaces.

[4] Jiang Y., Liu C., Li Y., Huang A. (2019). Stainless-Steel-Net-Supported
Superhydrophobic
COF Coating for Oil/Water Separation. J. Membr.
Sci..

[5] Divya, Jangir R. (2023). COF-Molecular Sieving
Membrane Fabrication and Applications for Dye Separation. ChemistrySelect.

[6] Ampong D. N., Effah E., Tsiwah E. A., Kumar A., Agyekum E., Doku E. N. A., Issaka O., Agyemang F. O., Mensah-Darkwa K., Gupta R. K. (2024). Advances and Challenges in Covalent Organic Frameworks
as an Emerging Class of Materials for Energy and Environmental Concerns. Coord. Chem. Rev..

[7] Li J., Cheng Z., Wang Z., Dong J., Jiang H., Wang W., Zou X., Zhu G. (2023). Ultramicroporous Covalent
Organic Framework Nanosheets with Functionality Pair for Membrane
C2H2/C2H4 Separation. Angew. Chem., Int. Ed..

[8] Karak S., Dey K., Banerjee R. (2022). Maneuvering
Applications of Covalent Organic Frameworks
via Framework-Morphology Modulation. Adv. Mater..

[9] Zhang Y., Ma L., Lv Y., Tan T. (2022). Facile Manufacture
of COF-Based Mixed
Matrix Membranes for Efficient CO2 Separation. Chem. Eng. J..

[10] Wang S., Wei X., Li Z., Liu Y., Wang H., Zou L., Lu D., Hassan Akhtar F., Wang X., Wu C., Luo S. (2022). Recent Advances in
Developing Mixed Matrix Membranes Based on Covalent
Organic Frameworks. Sep. Purif. Technol..

[11] Maji S., Ahmed S., Kaur-Sidhu M., Mor S., Ravindra K. (2023). Health Risks
of Major Air Pollutants, Their Drivers and Mitigation Strategies:
A Review. Air, Soil Water Res..

[12] Bharti R., Thakur A., Verma M., Sharma R. (2025). Environmental
and Health
Impacts of Air Pollution: A State of Art Review on the Strategy to
Control Air Pollution. AIP Conf. Proc..

[13] Baker, R. W. Membrane Technology and Applications, 3rd ed.; Wiley, 2012.

[14] Siagian U. W. R., Raksajati A., Himma N. F., Khoiruddin K., Wenten I. G. (2019). Membrane-Based Carbon Capture Technologies: Membrane
Gas Separation vs. Membrane Contactor. J. Nat.
Gas Sci. Eng..

[15] Guo Z., Wu H., Chen Y., Zhu S., Jiang H., Song S., Ren Y., Wang Y., Liang X., He G., Li Y., Jiang Z. (2022). Missing-Linker
Defects in Covalent Organic Framework Membranes for
Efficient CO2 Separation. Angew. Chem., Int.
Ed..

[16] Cheng Y., Ying Y., Zhai L., Liu G., Dong J., Wang Y., Christopher M. P., Long S., Wang Y., Zhao D. (2019). Mixed Matrix Membranes Containing MOF@COF Hybrid Fillers for Efficient
CO2/CH4 Separation. J. Membr. Sci..

[17] Wang Q., Guo Y., Long Y., Liu Y., Wang Z., Liu Y., Zhang X. (2024). Efficient Separation
of CO2/CH4 by Covering Ultrathin Ionic Liquid
Film on COF Membrane. J. Membr. Sci..

[18] Aydin S., Altintas C., Keskin S. (2022). High-Throughput Screening
of COF
Membranes and COF/Polymer MMMs for Helium Separation and Hydrogen
Purification. ACS Appl. Mater. Interfaces.

[19] Yu Y., Tang Y., Liu L., Wang Q., Yin C., Zhang K., Liu C., Ye Y., Zhao X., Gu T., Wang S. (2025). In Situ Synthesis of
TpPa COFs in Mixed Matrix Membranes
for Enhanced CO2 Separation. Ind. Eng. Chem.
Res..

[20] Cao X., Wang Z., Qiao Z., Zhao S., Wang J. (2019). Penetrated
COF Channels: Amino Environment and Suitable Size for CO2 Preferential
Adsorption and Transport in Mixed Matrix Membranes. ACS Appl. Mater. Interfaces.

[21] Shan M., Seoane B., Rozhko E., Dikhtiarenko A., Clet G., Kapteijn F., Gascon J. (2016). Azine-Linked Covalent
Organic Framework (COF)-Based Mixed-Matrix Membranes for CO2/CH4 Separation. Chem.—Eur. J..

[22] Biswal B. P., Chaudhari H. D., Banerjee R., Kharul U. K. (2016). Chemically Stable
Covalent Organic Framework (COF)-Polybenzimidazole Hybrid Membranes:
Enhanced Gas Separation through Pore Modulation. Chem.—Eur. J..

[23] Aguilar-Armenta G., Patiño-Iglesias M. E., Leyva-Ramos R., Leyva-Ramos R. (2003). Adsorption Kinetic Behaviour of Pure CO2, N2 and CH4
in Natural Clinoptilolite at Different Temperatures. Adsorpt. Sci. Technol..

[24] Amaral R. A., Habert A. C., Borges C. P. (2014). Activated Carbon
Polyurethane Membrane
for a Model Fuel Desulfurization by Pervaporation. Mater. Lett..

[25] Rezende C. G. F., Borges C. P., Habert A. C. (2016). Sorption
of Propylene and Propane
in Polyurethane Membranes Containing Silver Nanoparticles. J. Appl. Polym. Sci..

[26] Solouki
Bonab V., Manas-Zloczower I. (2017). Revisiting Thermoplastic Polyurethane,
from Composition to Morphology and Properties. J. Polym. Sci. B Polym. Phys..

[27] Hong T., Li Y., Wang S., Li Y., Jing X. (2022). Polyurethane-Based
Gas Separation Membranes: A Review and Perspectives. Sep. Purif. Technol..

[28] Cao X., Xu H., Dong S., Xu J., Qiao Z., Zhao S., Wang J., Wang Z. (2020). Preparation
of High-Performance and
Pressure-Resistant Mixed Matrix Membranes for CO2/H2 Separation by
Modifying COF Surfaces with the Groups or Segments of the Polymer
Matrix. J. Membr. Sci..

[29] Hoque B., Khan M. Y., Hanif A., Abdelnaby M., Helal A., Khan A., Usman M., Drmosh Q. A. (2024). Tailored
Design of CO2-Selective Mixed-Matrix Membranes Using Nitrile-Functionalized
COFs as 2D Nanofillers. J. Environ. Chem. Eng..

[30] Wijmans J. G., Baker R. W. (1995). The Solution-Diffusion
Model: A Review. J. Membr. Sci..

[31] Kandambeth S., Mallick A., Lukose B., Mane M. V., Heine T., Banerjee R. (2012). Construction of Crystalline
2D Covalent Organic Frameworks
with Remarkable Chemical (Acid/Base) Stability via a Combined Reversible
and Irreversible Route. J. Am. Chem. Soc..

[32] Martínez-Visus I. ´., Ulcuango M., Zornoza B., Coronas J., Téllez C. (2023). Green and
Fast Strategies for Energy-Efficient Preparation of the Covalent Organic
Framework TpPa-1. Chem.—Eur. J..

[33] Ikeda Y., Tabuchi M., Sekiguchi Y., Miyake Y., Kohjiya S. (1994). Effect of
Solvent Evaporation Rate on Microphase-Separated Structure of Segmented
Poly­(Urethane-Urea) Prepared by Solution Casting. Macromol. Chem. Phys..

[34] Sadeghi M., Semsarzadeh M. A., Barikani M., Ghalei B. (2011). Study on the
Morphology
and Gas Permeation Property of Polyurethane Membranes. J. Membr. Sci..

[35] Ge Y., Zhou H., Ji Y., Ding L., Cheng Y., Wang R., Yang S., Liu Y., Wu X., Li Y. (2018). Understanding Water Adsorption and
the Impact on CO2 Capture in Chemically
Stable Covalent Organic Frameworks. J. Phys.
Chem. C.

[36] Aksu G. O., Daglar H., Altintas C., Keskin S. (2020). Computational Selection
of High-Performing Covalent Organic Frameworks for Adsorption and
Membrane-Based CO2/H2 Separation. J. Phys. Chem.
C.

[37] Maia R. A., Lopes Oliveira F., Ritleng V., Wang Q., Louis B., Mothé
Esteves P. (2021). CO2 Capture by Hydroxylated Azine-Based Covalent Organic
Frameworks. Chem.—Eur. J..

[38] Wang S., Li X., Wu H., Tian Z., Xin Q., He G., Peng D., Chen S., Yin Y., Jiang Z., Guiver M. D. (2016). Advances
in High Permeability Polymer-Based Membrane
Materials for CO2 Separations. Energy Environ.
Sci..

[39] Robeson L. M. (2008). The Upper
Bound Revisited. J. Membr. Sci..

[40] Wang Y., Ren Y., Cao Y., Liang X., He G., Ma H., Dong H., Fang X., Pan F., Jiang Z. (2023). Engineering
HOF-Based Mixed-Matrix Membranes for Efficient CO2 Separation. Nanomicro Lett..

[41] Xiong S., Li L., Dong L., Tang J., Yu G., Pan C. (2020). Covalent-Organic
Frameworks (COFs)-Based Membranes for CO2 Separation. J. CO2 Util..

